# Reducing bias in secondary data analysis via an Explore and Confirm Analysis Workflow (ECAW): a proposal and survey of observational researchers

**DOI:** 10.1098/rsos.230568

**Published:** 2023-10-11

**Authors:** Robert T. Thibault, Marton Kovacs, Tom E. Hardwicke, Alexandra Sarafoglou, John P. A. Ioannidis, Marcus R. Munafò

**Affiliations:** ^1^ Meta-Research Innovation Center at Stanford (METRICS), Stanford University, Stanford, CA 94305-6104, USA; ^2^ School of Psychological Science, University of Bristol, Bristol, UK; ^3^ Doctoral School of Psychology, ELTE Eotvos Lorand University, Budapest, Hungary; ^4^ Institute of Psychology, ELTE Eotvos Lorand University, Budapest, Hungary; ^5^ Melbourne School of Psychological Sciences, University of Melbourne, Melbourne, Australia; ^6^ Department of Psychology, University of Amsterdam, Amsterdam, Noord-Holland, The Netherlands; ^7^ Meta-Research Innovation Center Berlin (METRIC-B), QUEST Center for Transforming Biomedical Research, Berlin Institute of Health, Charité – Universitätsmedizin Berlin, Berlin, Germany; ^8^ MRC Integrative Epidemiology Unit, University of Bristol, Bristol, UK

**Keywords:** blind data analysis, preregistration, ALSPAC, meta-research, open science, Explore and Confirm Analysis Workflow (ECAW)

## Abstract

*Background.* Although preregistration can reduce researcher bias and increase transparency in primary research settings, it is less applicable to secondary data analysis. An alternative method that affords additional protection from researcher bias, which cannot be gained from conventional forms of preregistration alone, is an Explore and Confirm Analysis Workflow (ECAW). In this workflow, a data management organization initially provides access to only a subset of their dataset to researchers who request it. The researchers then prepare an analysis script based on the subset of data, upload the analysis script to a registry, and then receive access to the full dataset. ECAWs aim to achieve similar goals to preregistration, but make access to the full dataset contingent on compliance. The present survey aimed to garner information from the research community where ECAWs could be applied—employing the Avon Longitudinal Study of Parents and Children (ALSPAC) as a case example. *Methods.* We emailed a Web-based survey to researchers who had previously applied for access to ALSPAC's transgenerational observational dataset. *Results.* We received 103 responses, for a 9% response rate. The results suggest that—at least among our sample of respondents—ECAWs hold the potential to serve their intended purpose and appear relatively acceptable. For example, only 10% of respondents disagreed that ALSPAC should run a study on ECAWs (versus 55% who agreed). However, as many as 26% of respondents agreed that they would be less willing to use ALSPAC data if they were required to use an ECAW (versus 45% who disagreed). *Conclusion.* Our data and findings provide information for organizations and individuals interested in implementing ECAWs and related interventions. *Preregistration*. https://osf.io/g2fw5 Deviations from the preregistration are outlined in electronic supplementary material A.

## 1. Introduction

Many published research findings are non-reproducible and potentially false or misleading [1–5]. These shortcomings may stem from a combination of researcher bias, publication bias, selective reporting of results, incomplete reporting of methods and other questionable research practices. These issues can lead to research waste, and ineffective or even harmful healthcare and policy interventions (e.g. [6]).

Some research disciplines have adopted standards to address these issues. For example, researchers conducting clinical trials regularly register outcome measures before enrolling participants, and they keep both participants and experimenters blind regarding group assignment. In cases where datasets already exist, however, researchers cannot easily adopt these practices. Researchers performing secondary data analyses generally have access to complete datasets without any blinding imposed on them. This may lead to exploration of diverse analyses and selective reporting based on the nature of the results.

### 1.1. Preregistration in the context of secondary data analyses

Increasing the uptake and effectiveness of preregistration for secondary data analyses may require a different approach from that for clinical trials. For example, the analytical space for secondary data analyses (e.g. of a longitudinal cohort dataset) is more vast than the analytical space for most clinical trials. Thus, the clinical trial standard of registering outcome measures, but no analysis plan, remains insufficient. Moreover, preregistration of secondary data analyses remains uncommon, and unlike for clinical trials (where the dataset does not yet exist), there is no guarantee that researchers performing secondary data analyses have not accessed the dataset. To extend the benefits of preregistration to secondary data analyses, we propose a workflow that necessitates the preregistration of an executable analysis script before researchers can access the full dataset.

### 1.2. Explore and Confirm Analysis Workflow (ECAW)

In this intervention that we propose, a data management organization that controls access to a pre-existing dataset would first provide access to only a subset of the data a researcher requests. After the researcher prepares an analysis script based on the subset of data and uploads it to a registry—where it will be openly accessible and permanent (e.g. to the Open Science Framework Registry)—the data management organization then provides access to the full dataset (e.g. via a secure data environment) and the researcher can proceed as they wish. The exact implementation (e.g. whether the data management organization runs quality checks on the analysis scripts) would depend on the preferences of the data management organization and the research community using their dataset (see box 1 for a hypothetical example).

Box 1. **A hypothetical example of an ECAW in practice.**

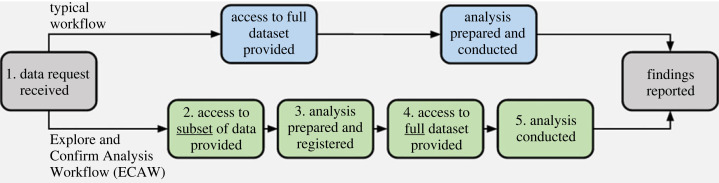

The overarching framework for ECAWs is depicted above. Within this framework, data management organizations would need to pre-specify several details for each step. Below, we provide a hypothetical example for illustrative purposes. The exact implementation of ECAWs would depend on the preferences of each data management organization and the community who uses their dataset.

1. A research team submits to a data management organization (i) a paragraph describing the analyses they want to run and (ii) a list of the variables they want to analyze.
2. The data management organization provides the research team with access to a subset of the data. For example, if the research team requests data for 15 variables collected from 10 000 participants, the data management organization will provide access to all 15 variables from a random subset of participants—say 1000.
3. The research team prepares an analysis script written in a common programming language (e.g. R or Stata) using the subset of data. They register this analysis script, the output from the script, and the paragraph they sent to the data management organization in Step 1, to www.osf.io/registries as an ‘Open-Ended Registration’. The researchers are free to run as many analyses as they would like on the subset of data. However, they should only register the analysis script they plan to use on the complete dataset.
4. The data management organization performs a basic quality check. They ensure that (i) the analysis script, the script output, and the paragraph are registered and (ii) the output contains a clear number of itemized results (e.g. as clinicaltrials.gov does for outcome measures). If the quality check fails, the data management organization asks the researchers to update their registration until it passes. The research team is then given access to a dataset with all the variables requested for all participants.
5. The researchers can proceed as they wish. They can run the registered analysis script on the complete dataset, make adjustments to the analysis if desired, or not proceed at all. The data management organization does not perform any additional check on what analyses were run. Regardless of what the research team decides, a permanent version of the planned analysis is available on the OSF Registry.

We call this process an Explore and Confirm Analysis Workflow (ECAW). Researchers may use the subset of data to generate hypotheses and/or to simply ensure that their intended analysis runs properly. ECAWs may increase the uptake of preregistration and provide assurance that the analyses were developed before the researchers observed the full dataset. Moreover, whereas conventional preregistrations often leave many degrees of freedom regarding exactly how the data will be analysed (e.g. [7]), ECAWs provide an executable analysis script. Researchers may also find ECAWs more agreeable than conventional preregistration because they allow for exploration. ECAWs would not fully solve publication bias, but could make it easier to detect because the registered analysis script would serve as a form of preregistration.

In short, ECAWs give researchers access to enough data to develop an informed analysis, but without compromising the confirmatory nature of a final analysis on the complete dataset.

### 1.3. Examples of ECAW-like workflows

One study probed the benefits and drawbacks of a workflow similar to ECAWs, but they provided a synthetic dataset rather than a data subset [8]. The researchers recruited 120 teams to analyze a single observational dataset and had half the teams preregister a written analysis plan and the other half prepare an analysis plan by drafting an analysis script based on a dataset with shuffled data for the variables of interest (i.e. the analysts were effectively blinded). Based on self-reports from the participants, the researchers found that the two workflows were comparable in terms of effort and that teams using blinded data analysis made fewer deviations from their analysis plan.

Some disciplines, such as particle physics, implement blind data analyses regularly [9,10]. A version of the ECAW workflow has also been successfully implemented by eight teams performing secondary data analysis on a dataset managed by the Psychological Science Accelerator group [11] and is currently being used for Registered Reports based on a large psychology dataset [12].

In medicine and clinical epidemiology, a similar workflow is implemented by the software platform OpenSAFELY (www.opensafely.org). This platform provides a dataset of simulated health records from which researchers can develop an analysis script. When ready, a researcher submits their analysis script which is automatically logged and made public in GitHub. The analysis then runs in a Trusted Research Environment (TRE) on data which is stored in the data centres where patients' records already reside (i.e. the data are not copied or moved). This workflow keeps individual health records hidden while also documenting all analyses run on the real data.

In this paper, we focus on ECAWs instead of synthetic datasets because they are likely more straightforward to implement for both data management organizations and researchers. The exploratory phase also allows researchers to view real data, which may help them generate hypotheses, especially if the hypotheses depend on moderators or confounders. If the ECAW is split into a test and validation dataset (with no overlapping data between the datasets), then this workflow can also serve as a form of replication, but at the cost of statistical efficiency (as proposed by Yarkoni & Westfall [13]). The strengths and weaknesses of various workflows for blind data analysis are outlined by Dutilh *et al*. [14].

### 1.4. Study objectives

Here, we present a descriptive and exploratory survey study. We had no hypotheses, but we did have two specific objectives. (1) To gain insights on the opinions and practices of researchers who already use pre-existing observational datasets, in regards to the trustworthiness and reproducibility of research. (2) To use these insights to inform future research—including a potential trial of ECAWs with the Avon Longitudinal Study of Parents and Children (ALSPAC)—on how data management organizations can encourage rigorous and reproducible research practices.

## 2. Methods

### 2.1. Participants

We sent an email to invite researchers on the mailing list for the UK-based ALSPAC to participate in an online survey (see electronic supplementary material B). We partnered with ALSPAC because they manage an oft-requested dataset and expressed interest in studying ways to ensure the research stemming from their dataset is rigorous. ALSPAC is ‘a transgenerational prospective observational study investigating influences on health and development across the life course. It considers multiple genetic, epigenetic, biological, psychological, social and other environmental exposures in relation to a similarly diverse range of health, social and developmental outcomes’ [15,16]. Thus, our invitation reached researchers that use observational data across the health and social sciences. The survey was open from 10 October 2022 to 1 November 2022. We sent two reminder emails, exactly one week and two weeks after the original email invitation. The mailing list comprises researchers whose email addresses were present on a proposal to access the ALSPAC dataset and included 1148 email addresses.

### 2.2. Survey

The survey contained 6 blocks and is available at https://osf.io/5h7gb. We developed the survey with feedback from the Principal Investigator and Executive Director of ALSPAC (Nicholas Timpson and Kate Northstone). We aimed to include as few questions as possible (to encourage a high response rate) while still garnering substantive information on whether ECAWs are relevant and acceptable to ALSPAC users.

Block 1 assessed the extent to which respondents believe that observational research using pre-existing data is trustworthy and reproducible (2 questions). Block 2 asked respondents how often they use practices related to transparency and reducing researcher bias, including preregistration, blinded data analysis, and sharing analysis scripts (5 questions). Between Block 2 and Block 3, the survey described ECAWs. Block 3 assessed the extent to which respondents believe that ECAWs would make observational research using pre-existing data more trustworthy and reproducible (2 questions). Block 4 directly asked respondents whether ALSPAC should run a study on ECAWs and whether they would participate (5 questions). Block 5 contained open-ended questions about the perceived benefits and drawbacks of ECAWs, reservations about ECAWs, whether additional incentives might be needed to use ECAWs, and suggestions for other research practices or policies that data management organizations could implement to improve research quality (3 questions). Block 6 asked participants how concerned they are about research quality, how many relevant studies they have published, what software they use for data analysis, and for any additional comments (4 questions). All rating-scale questions contained the response option ‘*I don't understand the question*’. The questions presented in figure 3 also contained the response option ‘*Unsure*’.

### 2.3. Analyses

**Figure 1.**
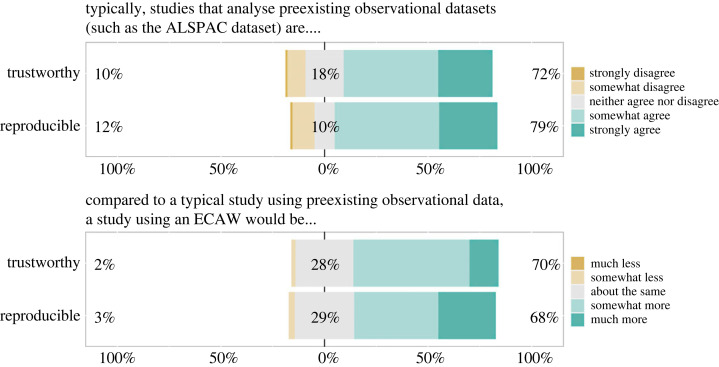
Responses to the survey questions on trustworthiness and reproducibility of observational research with pre-existing data and ECAWs. The survey defined trustworthy as ‘meaning that the results and conclusions of the publications are valid, reliable, rigorous, and accurate. That they merit trust’. The survey defined reproducible ‘in the sense that other researchers re-analysing the data with the same research question would produce similar results’. For each item, the number to the left of the data bar indicates the combined percentage for the responses depicted in any shade of brown/orange. The number in the centre of the data bar (grey) indicates the percentage of neutral responses. The number to the right of the data bar indicates the combined percentage for the responses depicted in any shade of green. The bar charts in the top panel had no missing responses or selection of the option *‘I don't understand the question’*. The bar charts in the bottom panel excluded responses of *‘I don't understand the question’* (*n* = 3; 2; respectively from top to bottom).

**Figure 2.**
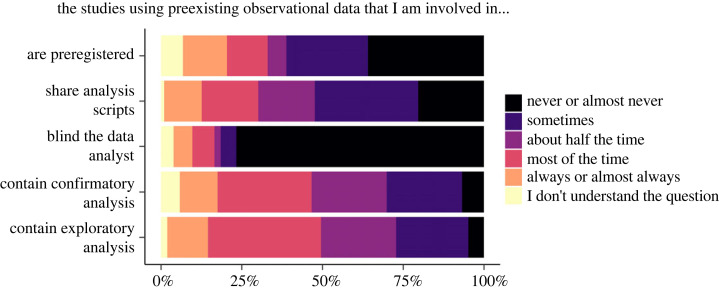
Responses to survey questions about the research practices of participants.

**Figure 3.**
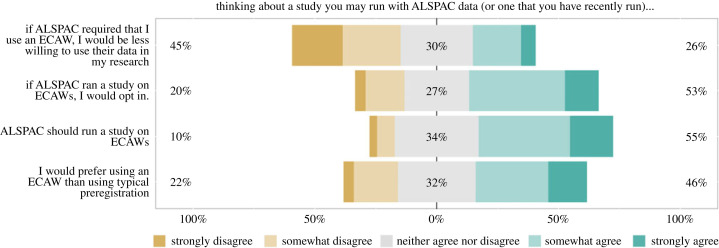
Responses to survey questions about using ECAWs. These bar charts exclude responses of *‘I don't understand the question’* (*n* = 0; 4; 1; 1), and responses of *‘Unsure’* (*n* = 2; 5; 6; 8). Agreement with the first question in this figure may be slightly inflated due to the format of the questions in this block. Respondents with a highly positive inclination towards ECAWs would be expected to disagree with the first question, but agree with the next three questions. Four respondents agreed with all four statements, suggesting they may have glazed over the word ‘less’ in the first question.^4^ Interpreting responses to the second and third question come with a degree of ambiguity as the survey did not specify what was meant by the term ‘study’.^5^

We present the results for closed-ended questions as prevalence rates or counts in figures 1–3 and electronic supplementary material C. When reporting percentages, we collapse together all positive responses on the rating scales (e.g. ‘*strongly agree*’ and ‘*somewhat agree*’) as well as all negative responses. The total number of responses differ among questions due to missing values and responses of ‘*I don't understand the question*’ and ‘*Unsure*’. We narratively synthesize the responses to the open-ended questions in table 1.^1^

**Table 1.** Recurring topics in responses to the open-ended survey questions. The survey included 4 open-ended questions with broad prompts regarding running a study on ECAWs, benefits and drawbacks of ECAWs, related research practices, and general comments. These questions received a total of 92 responses from 55 unique respondents. A complete list of responses are viewable in the open data. We synthesized the responses to open-ended questions into the 9 topics on the left side of this table. We divide these into three themes: (i) concerns about the acceptability of ECAWs, (ii) concerns that ECAWs will not have their intended impact, and (iii) alternative interventions that may achieve similar goals to conventional preregistration and ECAWs. On the right side of the table, we provide our reflection on each topic.

respondents’ comments^6^	our reflection
**concerns about acceptability**	
1. ECAWs would take too much time	compared to a study that is not preregistered, a study using an ECAW could very well take more time—especially at the beginning of the research process when preparing an analysis script. However, the advantages may outweigh the time commitment and further research could help understand this potential tradeoff. One survey suggests that psychology researchers believe that preregistration leads to longer project duration, but also higher research quality [17]. Moreover, compared to conventional preregistration, some evidence suggests that an ECAW-like workflow does not take more time [8]^7^
2. the subset of data would be insufficient for certain applications (e.g. for genome-wide association studies, rare outcomes, questions regarding small sub-samples, and imputation)	our survey suggested providing access to 10% of the dataset in the first ECAW stage. To develop an analysis for certain research questions, this subset of data may be insufficient. In these cases, a larger subset could be provided (e.g. 50%) and the final analysis could be run on the other 50% of the data, rather than the full dataset. Alternatively, the data management organization could create and provide access to a full-sized synthetic dataset
3. researchers will often have to change their analysis after seeing the full dataset	ECAWs do not stop researchers from changing their analysis. ECAWs make these changes transparent. This allows others to better assess risk of bias and calibrate their confidence in the study findings. As compared to conventional preregistration, some evidence suggests that an ECAW-like workflow leads to fewer deviations from the analysis plan [8]
4. researchers sometimes reuse the same dataset which they will already have full access to	ECAWs do not stop researchers from reusing the same dataset for new analyses. Although, as ECAWs were described in our survey, a research team could only use an ECAW once per dataset (because they will be exposed to the full dataset). Data management organizations could overcome this shortcoming by providing a secure data environment that publicly logs all analyses run, but never exposes the real dataset. Alternatively, they could provide access to only the subset of variables needed for a specific analysis. This would allow researchers to run another ECAW on the same larger dataset—if it focuses on variables not used in their previous ECAW
**concerns about impact**	
5. researchers can still *p*-hack/data-dredge when using ECAWs	as is the case for conventional preregistration, ECAWs discourage, but do not necessarily prevent *p*-hacking and data-dredging. ECAWs increase transparency by making these questionable research practices detectable
6. researchers may give findings from the subset too much weight (e.g. by not performing an analysis on the full dataset because it was null in the subset, or vice versa)	we hope that the instructions provided when implementing ECAWs make it clear that the subset of data is provided to help write an analysis script, but not to help decide which research questions to ask
**alternative interventions**	
7. data management organizations should require final analysis scripts to be shared (so that results are reproducible, and easy for other researchers to build on)	this policy could increase computational reproducibility. However, it may be difficult for data management organizations to implement and to ensure compliance. Whereas data management organizations can withhold access to the full dataset until an analysis script is registered, their influence is less direct at the publication stage
8. data management organizations should maintain a repository that outlines each research project conducted using their dataset (to reduce duplication and facilitate replication)	such a repository could be maintained in parallel with the use of ECAWs. It could also be used as a lighter touch intervention that may achieve some of the benefits we presume ECAWs would entail
9. data management organizations could make a synthetic version of their dataset openly available. That dataset could be used for ECAWs	several initiatives have employed synthetic datasets (e.g. OpenSAFELY [8,12]). On the one hand, creating a synthetic dataset requires more technical knowledge as compared to creating a data subset, and synthetic datasets can obscure the relationships between variables, which some researchers may dislike. On the other hand, they reduce the risk of participant re-identification and provide a full-sized dataset. All considered, synthetic datasets present a reasonable alternative to data subsets

## 3. Results

### 3.1. Survey completion

Of 1148 emails sent, 1094 went through and 54 bounced. The survey was completed 103 times and partially completed 20 times, leading to a response rate of 9% for complete surveys and 11% for at least partially complete surveys.^2^ The median time to complete the survey was 7.4 min (IQR: 4.6 to 13.1). This paper presents the results for complete surveys. Electronic supplementary material D presents the results with partially complete surveys included.

### 3.2. Participants

Respondents had published a median of 10 (IQR: 2 to 26) studies using pre-existing observational data (electronic supplementary material, figure C1). They reported using the following programming languages or software packages: R (*n* = 65), Stata (*n* = 48), SPSS (*n* = 17), SAS (*n* = 15), Python (*n* = 6), Mplus (*n* = 3), Bash (*n* = 2), MATLAB (*n* = 1), Nextflow (*n* = 1) and plink2 (*n* = 1) (electronic supplementary material, table C1).^3^ 62% (62/100) of participants reported being more concerned with research trustworthiness, bias, rigour and reproducibility compared to what they think of as a typical researcher who uses pre-existing observational data; 6% (6/100) reported being less concerned (electronic supplementary material, figure C2).

### 3.3. Survey results

Most respondents agreed that studies that analyse pre-existing observational datasets are trustworthy (72%; 74/103) and reproducible (79%; 81/103) (figure 1, top panel). At the same time, many believed that a study using an ECAW would be *more* trustworthy (70%; 70/100) and *more* reproducible (68%; 69/101) as compared to a typical study using pre-existing observational data (figure 1, bottom panel).

Over half of respondents reported that their studies using pre-existing observational data are preregistered never or almost never (36%; 37/103), or sometimes (25%; 26/103) (figure 2). About half reported sharing their analysis scripts never or almost never (20%; 21/103), or sometimes (32%; 33/103). A total of 77% (79/103) reported that they never or almost never blind the data analyst. Almost all respondents answered that they use both confirmatory (87%; 90/103) and exploratory (93%; 96/103) analyses at least sometimes.

A total of 26% (26/101) of respondents agreed (versus 45%; 45/101 who disagreed) that they would be less willing to use ALSPAC data if they were required to use an ECAW (figure 3). 53% (50/94) agreed (20%; 19/94 disagreed) that they would opt in if ALSPAC ran a study on ECAWs. 55% (53/96) agreed (10%; 10/96 disagreed) that ALSPAC should run a study on ECAWs. Finally, 46% (43/94) agreed (22%; 21/94 disagreed) that they would prefer using an ECAW than using conventional preregistration.

### 3.4. Exploratory analyses

Results can be explored interactively by following the instructions available at https://osf.io/wt6as. Based on the sample size of 103 participants and a lack of visually striking differences when exploring subsets of respondents, we do not report further on exploratory analyses.

## 4. Discussion

The survey results suggest that a trial of ECAWs with ALSPAC could be feasible for at least three reasons. First, ECAWs are possible because most respondents reported performing confirmatory analyses with analysis scripts written using programming languages. Second, ECAWs are relevant because many respondents reported limited use of other methods to improve rigour and reproducibility—including preregistration, sharing analysis scripts and blinding analysts. Moreover, although participants generally agreed that findings from observational research using pre-existing data are trustworthy and reproducible, they also believed that ECAWs would make research findings more trustworthy and more reproducible. Third, ECAWs appear relatively acceptable. For example, only 10% of respondents disagreed that ALSPAC should run a study on ECAWs, and 26% agreed that they would be less likely to use the ALSPAC dataset if they were required to use an ECAW.

The open-ended responses revealed interest in policies and interventions with similar goals to ECAWs. These include requirements for the sharing of final analysis scripts, a database of all ongoing studies that use a particular dataset, and openly available synthetic datasets.

### 4.1. Limitations

Given the response rate of 9%, our results represent the opinions of a select set of researchers who may be more interested and involved in reproducible research practices. Indeed, respondents themselves believed that they were more concerned about reproducibility than other researchers in their field (electronic supplementary material, figure C2). ECAWs may be less acceptable among non-responders and non-response may be a mark of lack of interest in the concept. Nonetheless, the absolute number of responders suggests that there is an audience of researchers who might be interested to pursue this approach.

The survey results are best understood as the initial thoughts of participants when introduced to the concept of ECAWs. The median response time was 7.4 min (IQR: 4.6 to 13.1) for a survey that included over 20 questions and a 500-word description of ECAWs. Thus, it is unlikely that respondents spent much time reflecting on ECAWs and their implications.

We invited researchers from the ALSPAC mailing list which includes researchers across the fields of health and social sciences, but we did not record the specific disciplines in which the respondents were active. Thus, while ECAWs may be more relevant to some disciplines, the present survey does not delve into the idiosyncrasies among disciplines.

Data management organizations could vary widely in their implementation of ECAWs. For example, they could perform checks on the analysis scripts to ensure they run and could require commented analysis scripts with a clear indication of the primary outcomes. Our survey results do not elucidate the best implementation of ECAWs.

### 4.2. Recommendations

Some data management organizations looking to implement ECAWs will need to consider a balance between updating their data access workflow and maintaining their user numbers, engagement and funding. This consideration will depend heavily on the structure of the data management organization. An organization managing data that is routinely collected somewhat regardless of its potential for use in research (e.g. electronic health records) may have leeway to test new workflows even if they impact user numbers. Other organizations—such as ALSPAC—coordinate ongoing data collection efforts that compound the value of their dataset and their continued operation remains contingent on funding cycles and data access fees. Even if ECAWs led to an increase in user numbers in the long term, a temporary decrease could preclude the cost recovery systems on which their staff rely. In a more extreme case, the likelihood of another successful funding cycle could be impacted and compromise the project's continuation. Funders and institutions interested in supporting these types of initiatives could alleviate concerns by providing targeted funding for testing these interventions and offering contingency funds to maintain cost recovery systems.

With these considerations in mind, an organization like ALSPAC may benefit from first leveraging the substantial number of respondents who would opt in to a study on ECAWs. Trialling ECAWs with this user group would allow organizations to collect data that may support more widespread implementation, including project completion time, study quality, and researcher satisfaction when using ECAWs. They could refine the ECAW pipeline with minimal concern about user numbers. In the situation that a data management organization is already considering implementing policies on preregistration, they may benefit from considering alternative workflows such as ECAWs, which many respondents deemed preferable to conventional preregistration. The open-ended responses to our survey also suggest some confusion around the purpose of ECAWs and the process of using them. A clear-cut module provided by data management organizations that explains the ECAW concept alongside step-by-step instructions could help address researchers' concerns preemptively and help them adopt this workflow.

The stakes are lower in cases where a static final dataset already exists and concerns about funding are absent. For example, researchers with an interest in rigorous analyses and who control access to a dataset have already employed ECAW-like workflows (e.g. [11,12]). Concerns about a reduction in user numbers and engagement may also be less relevant for datasets containing unique data. For example, a researcher trying to answer a question about health and development with ALSPAC data, may also be able to answer that question with another cohort dataset. However, a researcher trying to answer a question about the population of a specific country may need access to that country's census data, regardless of the workflow required by that data management organization. A final consideration is that user numbers and engagement may increase if researchers feel that ECAWs increase the trustworthiness of their findings and others come to associate research from datasets using ECAWs as more open and rigorous.

## 5. Conclusion

In this paper, we outlined a research workflow—ECAW—which necessitates certain open science practices and can be implemented by data management organizations. Responses to our survey provide information for organizations interested in developing and testing ECAWs and interventions with related goals.

## Data Availability

Data, data dictionaries, analysis script and materials related to this study are publicly available at https://osf.io/gmd3n/ [18]. The cleaned dataset used in our analyses is available at https://osf.io/vn695. The study protocol and materials were registered on 24 September 2022 at https://osf.io/g2fw5. Discrepancies between this paper and the registered protocol are outlined in electronic supplementary material A. To facilitate reproducibility, this paper was written by interleaving regular prose and analysis code using R Markdown. The relevant files are available in a Code Ocean container (https://doi.org/10.24433/CO.1558762.v1) [19] which recreates the software environment in which the original analyses were performed. This container allows this paper to be reproduced from the data and code with a single button press. Electronic supplementary material is available online [20].

## Footnotes

1 In other words, we provide a non-systematic qualitative summary of the open-ended responses. One author (R.T.T.) read through all the open-ended responses while writing down summarized versions of the responses and noting when responses made similar points. He then sorted responses with recurring and substantive content into distinct topics. He then categorized these topics into three overarching themes.

2 The ALSPAC mailing list has been maintained as a record of collaborators for many years and is constantly added to. The ALSPAC team only removes email addresses from their mailing list if someone explicitly requests this action. Thus, their list likely includes several email addresses that are no longer monitored. For example, we received one email reply stating that the recipient had not been active in research for 30 years. It is also possible that some researchers have multiple email addresses on the mailing list (e.g. because they moved institutions). These two factors may have deflated the response rate.

3 Participants could select multiple responses to this survey question.

4 Another four respondents agreed with at least the first and second question, which appear contradictory. We did not preregister these considerations. More careful wording of these questions could have circumvented the ambiguity in interpreting these seemingly contradictory responses.

5 We intended for these questions to ask about ALSPAC running a trial on ECAWs. However, due to the ambiguity around the terms ‘study’, some respondents may have interpreted this as a survey, focus group, feasibility or pilot type of study.

6 The text in the respondents' comments column is synthesized from multiple responses and/or paraphrased. They are not verbatim responses.

7 Sarafoglou *et al.*'s study used a pre-cleaned dataset. We are not aware of data that elucidate the time difference between ECAW-like workflows and conventional preregistration when starting from raw data.
